# Efficacy and Safety of Remdesivir in Adult Solid Organ Transplant Recipients: A Scoping Review

**DOI:** 10.3390/ph17060765

**Published:** 2024-06-11

**Authors:** Catherine Smith, Maria Eugenia Novara, Andrea Cona, Anna Dolcimascolo, Giulia Cancellieri, Francesca Mortillaro, Enrico Ottavio Giannini, Anna Carollo, Alessandra Mularoni, Alessio Provenzani

**Affiliations:** 1 School of Pharmacy, University of Pittsburgh, Pittsburgh, PA 5261, USA; cms330@pitt.edu; 2 Clinical Pharmacy Service, Mediterranean Institute for Transplantation and Advanced Specialized Therapies (ISMETT), 90127 Palermo, Italy; ccarollo@ismett.edu (A.C.); aprovenzani@ismett.edu (A.P.); 3 Infectious Diseases Unit, Mediterranean Institute for Transplantation and Advanced Specialized Therapies (ISMETT), 90127 Palermo, Italy; acona@ismett.edu (A.C.); amularoni@ismett.edu (A.M.); 4 School of Specialization in Hospital Pharmacy, University of Palermo, 90133 Palermo, Italy; annadolcimascolo993@gmail.com (A.D.); giulia.cancellieri@hotmail.it (G.C.); francesca.mortillaro92@gmail.com (F.M.); enrico.ottavio@gmail.com (E.O.G.)

**Keywords:** COVID-19, SARS-CoV-2, solid organ transplant, remdesivir, efficacy, safety

## Abstract

The SARS-CoV-2 infection has been associated with important mortality, particularly in immunocompromised patients, including solid organ transplant (SOT) recipients. Remdesivir (RDV) is an antiviral drug that has proven to be effective in reducing the replication of the virus in host cells, by which it may reduce the progression of symptoms and, consequently, the length of hospital stay and mortality. Randomized controlled trials have evaluated its use in the general population but never in SOT recipients. For the first time in this review, the safety and efficacy of RDV is evaluated in this specific population. The literature research was conducted using PubMed/MEDLINE and Scopus databases from 1 January 2020 to 24 November 2023, and 23 studies were analyzed. Although no clinical studies specifically evaluating this population have been conducted yet, RDV is likely safe for SOT patients when compared to the general population, so prescribers should consider utilizing RDV in SOT patients who are at high risk for progression to severe COVID-19. Future research will allow for the confirmation of the observed results and the acquisition of broader and clearer data regarding the safety and efficacy of the drug in this specific setting.

## 1. Introduction

Solid organ transplantation (SOT) has transformed the survival and quality of life of patients with end-organ dysfunction. SOT offers life-saving treatment for diseases considered terminal or those associated with a significant impairment on a patient’s quality of life [1]. Organ rejection is one of the most serious complications of any SOT, causing transplant patients to be on indefinite immunosuppression protocols to prevent it. The goal of various immunosuppression regimens is to prevent the proliferation and cytotoxic actions of T cells while also suppressing antibody production from B cells. Unfortunately, this makes patients treated with immunosuppressive drugs susceptible to infections, such as that caused by SARS-CoV-2 [1,2].

Over the last four years, the COVID-19 pandemic has changed the landscape of healthcare and led to significant mortality [3,4,5,6]. Several studies have been conducted to assess the impact of infection on SOT recipients, and the initial ones have highlighted an increased mortality rate compared to the general population. However, subsequent research has yielded different results [7]. In a recent review, it was observed that transplant patients instead showed an elevated risk of intensive care unit admission and the need for mechanical ventilation; this could be attributed to the presence of pre-existing comorbidities in these patients [8].

Remdesivir (RDV) is one of the drugs used for the treatment of COVID-19 infection. Although there is robust scientific evidence showing that it has been used successfully to treat COVID-19 in the general population [9,10], its safety and efficacy for SOT patients as a whole remains unclear [11,12], due to the lack of specific randomized control trials. Therefore, the purpose of this study is to review the existing literature assessing the efficacy of RDV in the SOT population infected with SARS-CoV-2 and its safety in this population. The information discussed for the first time in this review is important to clinical practice because it incorporates the most recent evidence in such a specific clinical context, considers pharmacodynamic interactions with maintenance medications, and provides new observations about RDV use for COVID-19, such as viral resistance genes.

## 2. Results

**Figure 1:**
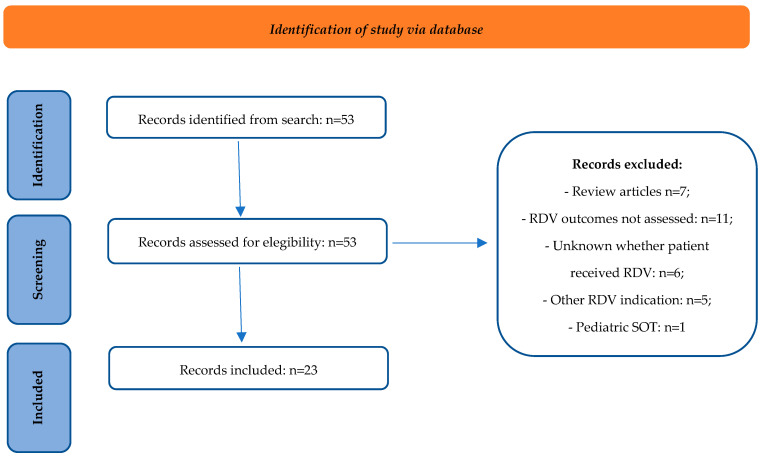
PRISMA flow diagram. RDV = remdesivir; SOT = solid organ transplant.

A total of 53 records were identified by applying the aforementioned research query and inclusion criteria and eliminating duplicate studies. Additionally, after applying the exclusion criteria, a full evaluation of the records was then conducted, and a total of 23 studies were assessed (Figure 1). Of these, there were 20 observational studies and three case reports. The main characteristics of the selected studies are summarized in Table 1.

### 2.1. RDV Safety in SOT Recipients

Although no randomized controlled trials have been conducted to explore the safety of RDV specifically in the SOT population, we can extrapolate data from observational studies in SOT recipients and RCT in the general population to make the most informed decisions possible when caring for these patients.

According to Sait AS et al., comparing early COVID-19 treatments to newer treatments, RDV does not appear to cause more significant adverse drug events in SOT patients, compared to the general population, and has not jeopardized patient safety with its use among SOT patients, compared to those who have not received a SOT [23].

One of the initial concerns of RDV use in transplant patients was organ toxicity, particularly the potential for elevation in transaminases and acute kidney injury (AKI). The use of RDV is no longer contraindicated in patients with renal dysfunction of less than 30 mg/dL [24,35,36,37,38,39,40,41], but there were initial concerns with its use in SOT patients, especially kidney recipients, due to the potential for the accumulation of the drug excipients [42]. Renal insufficiency is common in transplant patients, due to the concomitant use of a nephrotoxic immunosuppressant (i.e., tacrolimus), which has caused concern for many providers when selecting this agent in SOT recipients. Currently, there is no dose adjustment necessary for renal impairment. Elec F et al. analyzed 165 hospitalized patients with COVID-19 who received a kidney transplant; of these, 35 patients were treated with RDV, and no sign of nephrotoxicity was observed, with no difference in the incidence of AKI between the RDV and SOC groups (50% vs. 43%). In this study, immunosuppression was reduced by pausing mycophenolate mofetil or mycophenolic acid with or without adjusting calcineurin inhibitors. Tacrolimus was withdrawn in all patients receiving antiretrovirals and adjusted to maintain a trough level of 4–6 ng/mL in the other patients [24]. In a retrospective observational study, 57 KTRs were enrolled to evaluate the safety of RDV, and the outcomes were measured as AKI recovery, liver function test abnormalities, other side effects, graft loss, and death. The cohort of KTRs had a wide renal range function (59–21 mL/minutes) with a 66.6% baseline AKI, and RDV was administered to all patients. Most of the KTRs had a complete recovery at the 28-day follow-up, and only one case of graft loss was reported in a patient with baseline chronic graft dysfunction. Furthermore, no liver function derangements or major adverse events were observed, and no dosage adjustments of calcineurin inhibitors were necessary [25]. Tatapudi et al. conducted a retrospective study in India on 20 KTRs with SARS-CoV-2 infection. A total of 12 patients of this cohort were received (moderate or severity illness): 8 patients required oxygen with a mask, and 4 had escalated oxygen requirements. The treatment involved the use of RDV for 5–10 days and dexamethasone 6 mg for 6 days. The authors did not observe any deterioration of hepatic or renal function, and the latter remained stable (eGFR above 30 mL/min/1·73 m^2^). Tacrolimus dosage was adjusted by monitoring trough levels and maintaining it at 4–6 ng/mL, and there was no nephrotoxicity due to supratherapeutic levels [29].

Buxeda A et al. conducted a multicenter a study in which 51 hospitalized KTRs with COVID-19 were enrolled and treated with RDV. AKI was present in 22.7% of the patients, and after RDV, it happened in 11.7%, but no patients required the discontinuation of treatment because of renal impairment. Furthermore, the level of tacrolimus was analyzed before and after RDV use. In 22 patients who maintained the treatment, a significant decrease in tacrolimus levels after treatment was observed, although the calcineurin inhibitor dose was reduced in 11 of 22 patients at admission. Regarding the liver function, the authors did not observe a significant ALT/AST increase [31].

An elevation in liver transaminases has also been reported, which is concerning, especially for liver transplant recipients. This side effect is typically transient and does not commonly lead to RDV discontinuation [20]. According to the studies included in our search, no significant increase in liver enzymes was found in patients who received RDV [9,11,13,14,21,22]. Jamir I et al. did not observe an increase in transaminases in a liver transplant recipient who received RDV for COVID-19 one month after transplant. In another study, Jamir I et al. showed that there was no significant difference in adverse drug reactions to RDV in immunocompromised versus immunocompetent patients [32]. Shafiekhani M et al. demonstrated that there was no statistically significant difference in transaminases or reduced eGFR in patients receiving different treatments for COVID-19; however, there were numerically more incidents of decreased eGFR in RDV than other treatments [22].

Though RDV is known to cause incidents of AKI and elevation in liver enzymes, it seems that these do not amount to statistically significant differences, compared to their occurrence in non-transplanted patients and perhaps even compared to other treatments for COVD-19.

Some authors reported a significant increase in the serum concentration of tacrolimus when using RDV. The correct management of tacrolimus levels is critical both for the prevention of graft rejection and for the avoidance of excessive immunosuppression and drug toxicities [19,42,43]. Habeeb E et al. showed that levels significantly increased, peaking on day 3 of RDV therapy and returning to normal levels 10 days after the discontinuation of RDV. No adverse events resulted from this increase in this trial. The clinical significance of this is unknown, but theoretically, it could lead to excessive immunosuppression and resulting complications, such as superinfections, post-transplant lymphoproliferative disorder (PTLD), and reactivation of latent viruses. This potential adverse event should be considered when utilizing RDV in any patient receiving tacrolimus and, through level, should be closely monitored. However, compared to other antivirals, including nirmatrelvir/ritonavir, RDV has fewer drug interactions with immunosuppressants [19].

### 2.2. RDV Efficacy in SOT Recipients

SOT patients are a unique population managed on very strict regimens of medication. When a SOT recipient becomes infected with SARS-CoV-2, it is critical to manage them properly to protect the patient from severe complications of COVID-19 and to not reduce the effects of their maintenance medications. In this section, we will assess the efficacy of RDV in SOT patients for COVID-19, demonstrated by the literature in our search.

In a retrospective study conducted in the nephrology unit of a tertiary care hospital in New Delhi, Jasuja et al. observed a significant mortality in KTRs with COVID-19 infection treated with RDV: the mortality rate was 77.8%, similar to that observed in patients who received convalescent plasma and dialysis. However, RDV was used in hospitalized patients with severe infections who were unable to maintain oxygen saturation or hemodynamic stability with standard care [30].

During the Omicron BA.2 wave, Solera JT et al. conducted a prospective cohort study which enrolled 192 SOT recipients. Patients received either three doses of RDV as an outpatient if they were not hypoxemic and if they could start treatment within 7 days of COVID-19 symptom onset. Most of the patients did not receive any previous outpatient treatment; only seven patients received nirmatrelvir/ritonavir, and one received bebtelovimab. The number of SOT recipients treated with RDV needed to prevent one hospitalization was 15.2. Early outpatient RDV treatment was also associated with a decreased rate of hospitalization, compared to those who did not receive RDV [13]. A retrospective study from Colaneri M et al., conducted in 24 SOT recipients at an infectious disease clinic from December 2021 to February 2022, also looked specifically at outpatient RDV efficacy for COVID-19. Patients who received treatment other than RDV were excluded; therefore, patients who received RDV (*n* = 7) or those who had not received treatment (*n* = 17) were included. The group of patients who did not receive RDV was asymptomatic at the point of COVID-19 diagnosis or had an eGFR < 30 mL/min. Among 24 patients, 8 patients were already hospitalized for non-COVID-19-related reasons, and among them, only 1 patient was treated with the 3-day RDV. No patient required mechanical ventilation, and one patient died. No outpatients were hospitalized or showed a worsening of the disease. In this study, RDV treatment did significantly reduce hospitalization rates or the worsening of COVID-19 symptoms [16]. From these studies, RDV appears to be an appropriate option for early outpatient treatment to prevent more severe disease in SOT patients.

A retrospective study from Shafiekhani M et al. (2021) enrolled 245 transplant recipients, primarily including kidney and liver recipients, mostly within six months of transplant. RDV was administered to COVID-19 patients hospitalized for at least 48 h. In this population, RDV administration was associated with the reduced length of hospital and ICU stays and mortality, compared to other studies in which outdated treatment options, such as interferon and lopinavir/ritonavir, were described. Based on the studies included in our review, RDV seems to be equally effective in immunocompromised hosts, including SOT patients, as in the general population [22]. In a retrospective cohort study, 165 hospitalized patients with COVID-19 who received a kidney transplant were enrolled to evaluate the impact of RDV on overall mortality, ICU mortality, and renal functional outcome. This population was divided into RDV and standard of care (SOC) groups. ICU mortality was significantly reduced in the first group (39% vs. 83%), despite the number of patients with severe COVID-19 being higher in the RDV group (42% vs. 14%,) [24]. Meyyappan J et al. conducted a prospective study in KTRs with mild–moderate or severe COVID-19. In the last group, the mortality was similar between those who received and did not receive RDV, but the surviving patients received the drug early during the first week of illness. Furthermore, deaths in the first group were not observed, suggesting a benefit for mortality in the early use of RDV [26]. In an observational study conducted on a small cohort of KTRs, RDV was administered for the treatment of COVID-19 infection. All patients exhibited AKI and required oxygen support, with two patients necessitating mechanical ventilation. Only one patient succumbed to severe intestinal bleeding. The administration of RDV did not lead to any worsening of renal function or the need for hemodialysis. A slight increase in liver enzymes was observed. The authors of this study noted a certain level of drug safety, although the small number of enrolled patients did not allow for definitive conclusions [27]. In another small study conducted on heart transplant recipients with COVID-19, RDV was administered to those hospitalized with moderate infection. Furthermore, one patient received RDV as a second-line therapy when the infection became severe and after failure with molnupiravir. The administration of the drug proved effective in treating the infection, even with delayed administration (after two weeks). None of the patients required mechanical ventilation or intensive care unit admission; additionally, no episodes of rejection were observed. Although the data are limited, it appears that RDV may be a viable treatment option [28].

Regarding the time distribution of SARS-CoV-2 infection, two studies examined the outcomes of COVID-19 in SOT patients in two different time periods: March–May 2020 and June–November 2020 (Sait et al.) and 1 March–19 June 2020 and 20 June–31 December 2020 (Heldman, et al.). Between these periods, the emergency use authorization of RDV for COVID-19 in the United States was approved, and the use of steroids in patients requiring supplemental oxygen was recommended. In both studies, very few patients received RDV in the first period, and the majority of patients (42.9% and 53%, respectively) received RDV in the second time period. The study by Sait AS et al., which included a smaller cohort, did not find a statistically significant difference in mortality between the groups but did conclude that preexisting graft dysfunction was a significant risk factor for mortality [23]. The second and larger study by Heldman MR et al., which included 973 SOT patients, found that mortality significantly decreased in the second period; however, ICU admission did not significantly differ between groups [21,44].

The COVID-19 vaccines may have influenced the observed outcomes, but their commercialization occurred only towards the end of the indicated period. Therefore, the increased use of RDV appears to have significantly impacted the reduction in all-cause mortality. Additionally, it is worth considering that while the initial vaccines demonstrate efficacy primarily against the prevalent genetic variant at the time of vaccination (Delta), RDV seems effective against all different variants. This could indicate its efficacy not only in the general population but also in SOT patients [45,46,47].

A case-control study was conducted between September 2020 and May 2021 comparing outcomes for RDV versus standard of care in 100 patients hospitalized with COVID-19. There were 19 lung transplant recipients included in this study, as well as 6 patients with other transplanted organs. The cohort of SOT patients was divided into those treated with RDV (RDV-TX) and patients treated with standard care alone (SOC-TX). All patients in the RDV-TX were in supplemental oxygen therapy, while only one SOC-TX patient was on required oxygen, demonstrating that overall, a higher severity of disease led to more RDV use. The length of hospital stay was longer in RDV-TX patients as compared to non-transplanted patients who received RDV. However, when compared to the SOC-TX group, RDV was associated with greater 60-day survival, and decreased ICU admission, noninvasive ventilation, and death, even though statistically significant, were not reached for these outcomes. Another major consideration for these results is that all six patients who died in this study were lung transplant recipients. This type of transplantation seems to carry a higher risk of negative outcomes from COVID-19 than other organs. This study supports that although RDV is likely safe in SOT recipients for COVID-19, demonstrated by a lack of adverse events in RDV compared to standard of care, it may not be as effective as for the general population, particularly for those who have received a lung transplant [15]. A retrospective study from Zimmermann J et al. examined six lung transplant recipients where RDV was utilized in five of these patients. Four of the survivors received RDV. One patient, who received RDV as well as many other medications, died in this study, but the five other patients all survived [18]. This literature suggests the particular severity of COVID-19-related illness in lung transplant recipients, as well as the potentially less beneficial effect of RDV on this subpopulation.

One prospective cohort study from Rajme-López S et al. evaluated RDV use versus no RDV in 126 immunocompromised hosts, including 31 SOT recipients. Interestingly, SOT as a variable was not associated with a difference in mortality or hospitalization, compared to the total population. In this study, the RDV group showed significantly less mortality and hospitalization than those who were not treated. This was limited by the fact that the RDV group’s mean age was 7 years younger than that of the group that did not receive RDV [14].

Various case reports demonstrate the place in therapy of RDV in the SOT population. For example, one case study reported that one liver transplant patient who tested positive for SARS-CoV-2 received RDV for treatment. This patient was managed as an inpatient, and then was discharged home with no further complications of the disease [11]. Another liver transplant recipient received 7 days of RDV for SARS-CoV-2 re-infection. This patient was 10 years post transplantation, only experienced a mild disease, and survived [34]. Another liver transplant recipient experienced benefit from RDV utilization for his SARS-CoV-2 infection that he developed on day 21 post-transplantation. His case was severe, evolving into COVID-19 pneumonia and requiring extensive treatment. He was treated with 5 days of RDV alongside 3 days of convalescent plasma, and after 10 days, he was discharged from ICU. Eventually, he was discharged home and successfully treated with RDV immediately following his transplantation [32]. For liver transplant patients in these case studies, RDV seems to be beneficial. Lung transplant patients, however, seem to experience a higher severity of COVID-19. A case report article examined eight lung transplant recipients treated for COVID-19. The two most severe cases utilized RDV as part of a clinical trial, yet both of these patients died. Importantly, both of these patients were infected immediately following their lung transplantation [17]. These case reports demonstrate that not all SOT recipients have the same risk for severe disease and that RDV may not be as effective in lung transplant recipients.

Finally, an emerging concern of RDV use in immunocompromised patients, such as SOT recipients, is the emergence of viral resistance variables. Certain genes have already been identified in SOT recipients with multiple persistent SARS-CoV-2 infections, causing RDV to be less effective in these patients [33]. Providers should keep this in mind when utilizing RDV in these patients, but until there is evidence to suggest otherwise, they should not shy away from using RDV in SOT patients who have previously received RDV for a persistent SARS-CoV-2 infection

## 3. Discussion

SOT patients require a complex pharmacological management foundation, which becomes even more complicated in the presence of SARS-CoV-2 infection. These patients typically receive high doses of immunosuppressive drugs, which theoretically could render them more susceptible to complicated SARS-CoV-2 infections, as seen with other infections [48,49]. It is interesting to note that SOT patients may not have a higher mortality rate from COVID-19, due to their immunocompromised state. In fact, their immunosuppression may protect them from one of the deadly complications of COVID-19, cytokine release syndrome, which can cause severe respiratory complications [43]. RDV is an antiviral drug prescribed under EMA authorization for COVID-19, which appears to benefit the general population. For the first time, this review describes literature data on the efficacy and safety of RDV in the specific context of SOT patients. Although no clinical studies specifically evaluating this population have been conducted yet, data have been extrapolated from studies including immunocompromised patients, including SOT recipients. The inability to generalize the information contained in the included studies on the population of interest has not allowed for a systematic literature review, representing a limitation of this study. Nevertheless, it is possible to draw some conclusions regarding the role of RDV therapy in the SOT population. RDV may cause AKI and increased transaminases [50,51], even in SOT patients; however, these adverse effects are not significantly more common in SOT recipients, compared to the general population. Furthermore, reported increases in transaminases resolved without treatment and did not require RDV discontinuation. Therefore, RDV is safe to use in SOT patients [9,11,13,14,21,22]. Moreover, from the included studies, it appears that immunosuppressive drugs used for the prevention of rejection (such as tacrolimus) can be concurrently used with RDV, with the only precaution being to monitor blood levels [24,25,29,31].

Currently, SOT patients are likely to benefit from the use of RDV, compared to other treatments. This drug may not be as effective in SOT recipients as in the general population, but considering its favorable safety profile in SOT patients, it should be considered for use in these patients. Some data have shown that SOT patients receiving outpatient RDV infusion early in their course of SARS-CoV-2 infection have their hospitalization rates reduced. Other data indicate a decrease in mortality with the use of RDV in these patients [44,52]. Therefore, with clinical discretion, SOT recipients should be eligible for RDV treatment for COVID-19, both as an early outpatient treatment and as hospital management. Thus, despite the limited data collected, the gathered information may support the use of RDV in SOT recipients.

## 4. Materials and Methods

### 4.1. Data Source

C.S. and M. E. N. independently searched the PubMed/MEDLINE and Scopus online databases from 1 January 2020 to 24 November 2023 using the following search strategy: ((COVID-19) OR (SARS-CoV-2)) AND (remdesivir) AND ((solid organ transplant) OR (solid organ transplantation)). The protocol for this study was designed following the Preferred Reporting Items for Systematic Reviews and Meta-Analyses (PRISMA) Extension for Scoping Reviews (PRISMA-ScR) [53].

### 4.2. Study Eligibility Criteria

The research was conducted by applying the following inclusion criteria:

- Case reports, observational studies, and randomized control trials in the English language from 1 January 2020 to 24 November 2023;
- Studies of efficacy and safety describing the use of RDV in SOT recipients with COVID-19;
- Population of any ethnic group aged ≥18 years old.
- Exclusion criteria:
- Review articles;
- Studies that did not report or discuss the efficacy and safety of RDV;
- Pediatric studies;
- Studies involving the use of RDV in the prophylaxis of transplantation from a SARS-CoV-2-positive donor.

The results were verified by two authors separately, resolving any discrepancies through discussion. No duplicate articles were identified, so all records were screened for eligibility (Figure 1).

## 5. Conclusions

RDV is an antiviral medication that is beneficial for the general population infected with SARS-CoV-2, as proven by RCTs, systematic reviews, and meta-analyses. There are no randomized controlled trials evaluating its use in SOT recipients or sub-analyses of this subgroup in available RCTs. Therefore, in lieu of robust data, this scoping review of current evidence regarding the use of RDV in SOT recipients with COVID-19 aims to guide providers in prescribing RDV to SOT patients. Based on many observational studies, cohort studies, and case reports, RDV is likely safe and effective in SOT patients to the same extent as it is in the general population. Adverse effects do not seem to differ significantly from the general population, and it is unlikely that RDV jeopardizes graft function. Prescribers should consider utilizing RDV in SOT patients who are at high risk for progression to severe COVID-19. However, future large-scale studies focusing on the safety and efficacy of RDV in SOT patients are needed to further strengthen this evidence.

## Figures and Tables

**Table 1:** Main characteristics of the selected studies.

Article	Types of Clinical Study Designs	Population	Safety Studies	Efficacy Studies
Solera JT, et al. [13] (2023)	Observational study	192 SOT patients: 80 kidney, 37 lung, 36 liver, 12 heart, 27 unspecified combined transplants.	No	Yes
Rajme-López S, et al. [14] (2022)	Observational study	126 high risk patients. Includes 31 SOT recipients: Unspecified transplant types.	No	Yes
Yi SG, et al. [11] (2020)	Observational study	21 SOT patients: 12 kidney, 3 liver, 2 lung, 1 heart/lung, 1 liver/kidney, 1 heart/kidney, 1 kidney/pancreas.	No	Yes
Fesu D, et al. [15] (2022)	Observational study	25 SOT patients: 19 lung, 3 kidney, 2 liver, and 1 heart recipient.	Yes	Yes
Colaneri M, et al. [16] (2022)	Observational study	24 SOT patients: 19 kidney, 2 liver, 2 heart, 1 lung.	No	Yes
Myers CN, et al. [17] (2020)	Observational study	8 SOT patients: 8 lung transplant recipients.	No	Yes
Zimmermann J, et al. [18] (2022)	Observational study	6 SOT patients: 6 lung transplant recipients.	No	Yes
Habeeb E et al. [19] (2023)	Observational study	61 SOT patients: 27 kidney, 20 heart, 14 lung.	Yes	No
Biscarini S, et al. [20] (2022)	Observational study	143 immunocompromised patients, including 42 SOT patients: 23 kidney, 12 liver, 5 lung.	Yes	Yes
Heldman MR, et al. [21] (2022)	Observational study	973 SOT patients: 608 kidney, 138 liver, 120 heart, 104 lung, 3 other.	No	Yes
Shafiekhani M et al. [22] (2021)	Observational study	245 SOT patients: 143 kidney, 95 liver, 1 pancreas/kidney, 3 bowel, 3 multivisceral.	Yes	Yes
Sait AS, et al. [23] (2022)	Observational study	77 SOT patients: 41 kidney, 16 liver, 12 lung, 1 hand, 2 kidney/liver.	Yes	Yes
Elec F, et al. [24] (2022)	Observational study	35 SOT patients: 35 kidney transplant recipients.	Yes	Yes
Meshram HS, et al. [25] (2021)	Observational study	57 SOT patients: 57 kidney transplant recipients.	Yes	No
Meyyappan J, et al. [26] (2022)	Observational study	104 SOT patients: 104 kidney transplant recipients.	No	Yes
Latief M, et al. [27] (2022)	Observational study	7 SOT patients: 7 kidney transplant recipients.	No	Yes
Nowak A, et al. [28] (2023)	Observational study	5 SOT patients: 5 heart transplantations.	No	Yes
Tatapudi RR, et al. [29] (2021)	Observational study	20 SOT patients: 20 kidney transplant recipients.	Yes	No
Jasuja S, et al. [30] (2021)	Observational study	67 SOT patients: 67 kidney transplant recipients.	No	Yes
Budexa A, et al. [31] (2021)	Observational study	51 SOT patients: 51 kidney transplants recipients.	Yes	No
Jamir I, et al. [32] (2020)	Case report	1 SOT patient: 1 liver transplant recipient.	Yes	Yes
Hogan JI, et al. [33] (2023)	Case report	2 SOT patients: 2 kidney transplant recipients.	No	Yes
Mohseni M, et al. [34] (2021)	Case report	1 SOT patient: 1 liver transplant recipient.	No	Yes

SOT = solid organ transplant.

## Data Availability

Data sharing is not applicable.
